# Video‐Assisted Thoracoscopic Removal of a Mediastinal Mass in a Newborn: A Case Report

**DOI:** 10.1111/jpc.70187

**Published:** 2025-09-06

**Authors:** Carlo Maria Ferlini, Alessia Arossa, Claudia Codazzi, Stefano Ghirardello, Simonetta Mencherini, Luca Lungarotti, Giovanna Riccipetitoni, Mirko Bertozzi

**Affiliations:** ^1^ Department of Clinical, Surgical, Diagnostic and Pediatric Sciences University of Pavia Pavia Italy; ^2^ Pediatric Surgery Unit, Department of Maternal and Child’s Health Fondazione IRCCS Policlinico San Matteo Pavia Italy; ^3^ Obstetrics and Gynaecology Unit, Department of Maternal and Child's Health Fondazione IRCCS Policlinico San Matteo Pavia Italy; ^4^ Pediatrics Unit, Department of Maternal and Child's Health Fondazione IRCCS Policlinico San Matteo Pavia Italy; ^5^ Neonatal Intensive Care Unit, Department of Maternal and Child's Health Fondazione IRCCS Policlinico San Matteo Pavia Italy; ^6^ Anesthesia and Post‐Surgical Intensive Care Unit, Cardio‐Thoracic‐Vascular Department Fondazione IRCCS Policlinico San Matteo Pavia Italy; ^7^ Pediatric Radiology, Department of Radiology Fondazione IRCCS Policlinico San Matteo Pavia Italy

## 1 Introduction

Mature teratoma is a benign germ cell tumour most commonly arising in the ovaries, testis or sacrococcygeal region. The mediastinum is a less common localisation, with mature teratomas accounting for roughly 10% of all mediastinal tumours across all age groups [1]. They are most frequently found in the anterior mediastinum, arising from the thymic gland. Symptoms can vary depending on age: severe respiratory distress in newborn and younger children, chest pain or facial fullness due to impaired venous return in older children, incidental diagnosis in some instances.

Their great variability in presentation and unpredictability in outcome with likelihood of severe complications is the rationale for surgical indication.

In most cases in the current literature, resection is accomplished by open surgery. Video‐assisted thoracoscopic surgery (VATS) for mediastinal teratoma in children, particularly in newborns, is relatively less employed, even if it appears safe and effective in selected patients. We describe the case of a newborn with prenatal diagnosis of anterior mediastinal mass suggestive of benign teratoma who underwent a successful thoracoscopic removal.

This case report has been reported in line with the SCARE Criteria [2].

## 2 Case Report

A 34‐year‐old, 36‐week pregnant woman was referred to our prenatal tertiary centre with an ultrasound diagnosis of foetal pleural effusion and a 2‐cm wide, non‐vascularised thoracic solid lesion. The patient did not undergo the first trimester screening for aneuploidy; the 20‐week ultrasound was reportedly normal. Family history was unremarkable. The pregnancy was complicated by gestational diabetes treated with dietary measures and by J/k isoimmunisation (titre 1:16). Foetal biometry resulted at the 50th percentile and the middle cerebral artery peak systolic velocity was within the normal range.

**FIGURE 1:**
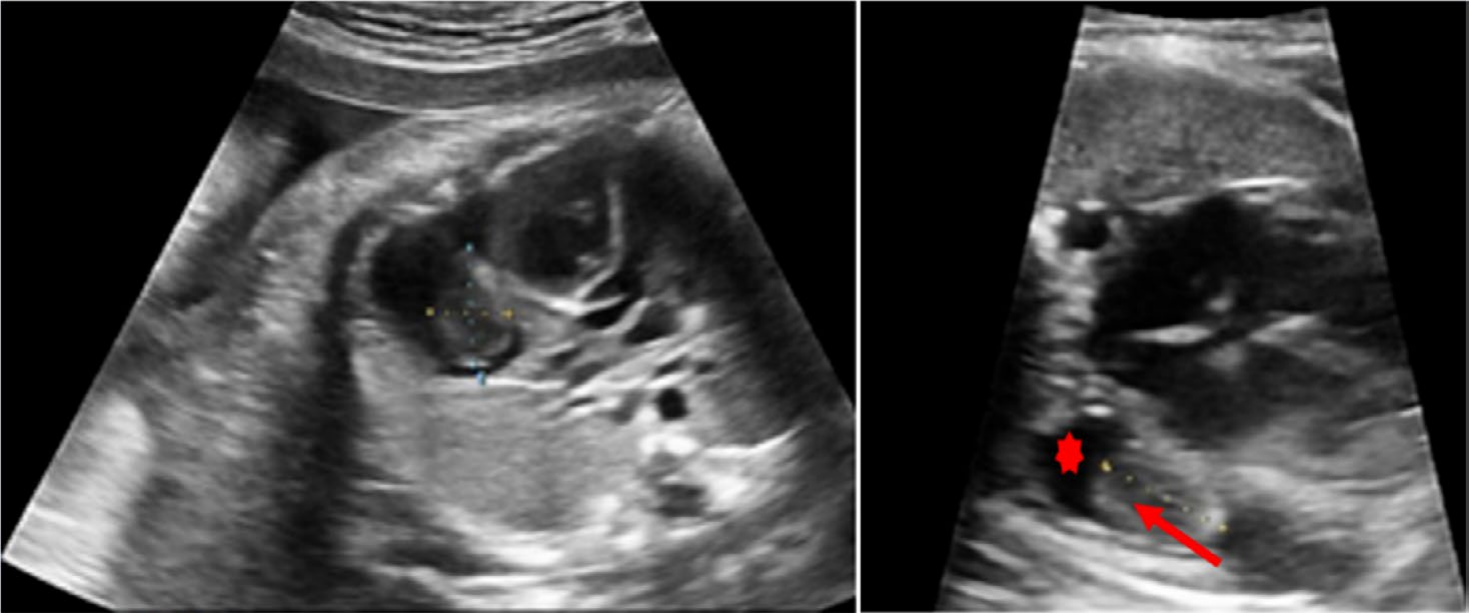
Foetal cardiac ultrasound. The non‐vascularised, heterogeneous thoracic formation skirted by (arrow) the lateral pericardial effusion (asterisk).

We performed a foetal cardiac sonography which showed a non‐vascularised, heterogeneous formation laterally and posteriorly to the right atrial wall. It appeared partly hyperechoic and partly iso‐hypoechoic and moved jointly with atrial movements, with a non‐circumferential right lateral pericardial effusion skirting around it (Figure 1).

**FIGURE 2:**
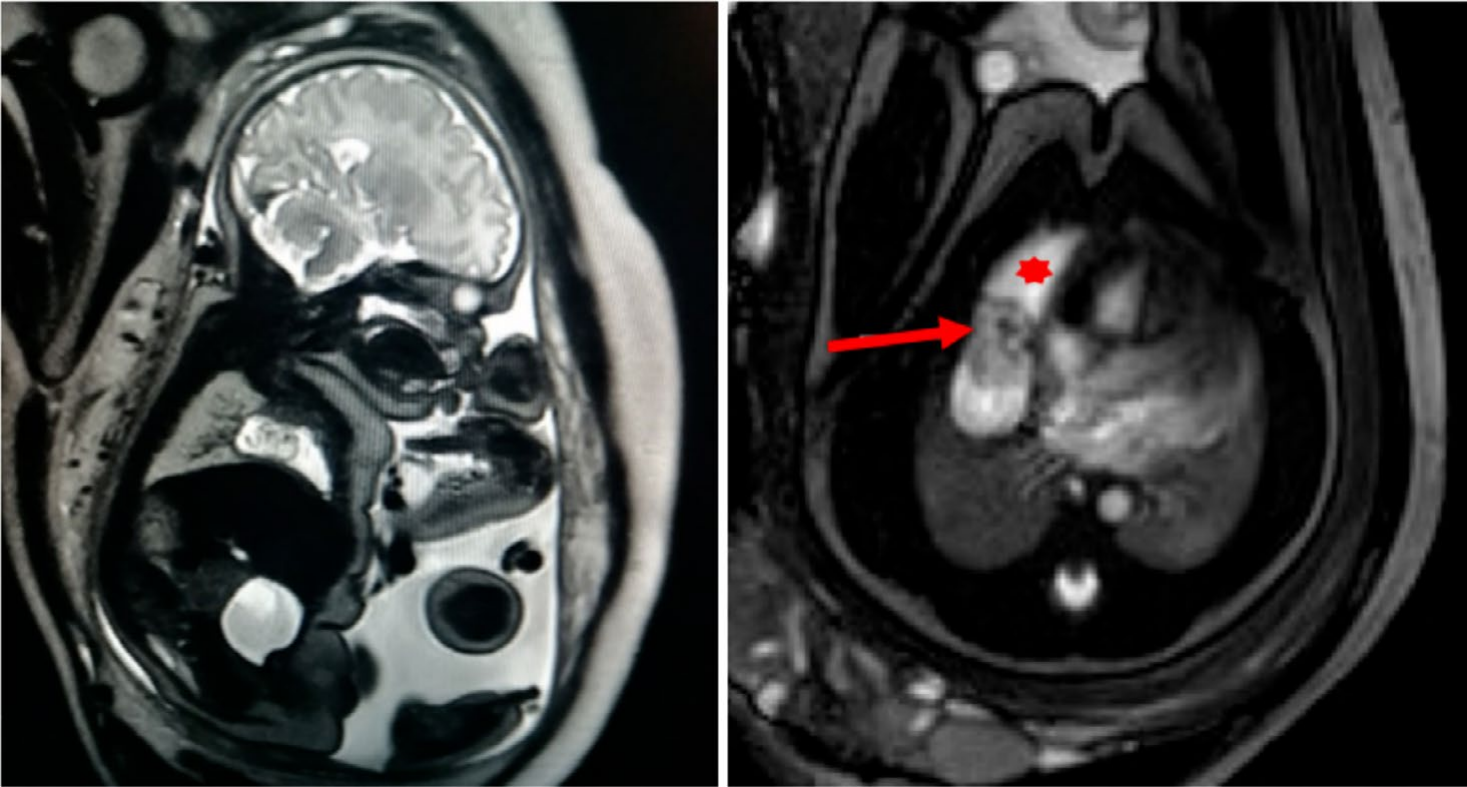
Foetal MRI. Axial balance turbo field echo (b‐TFE) image shows a well‐defined cystic lesion (asterisk) lying in the right anterior cardiophrenic angle, with an inhomogeneous, nodular, solid low‐signal lesion within it (arrow). No concurrent pericardial effusion is noted.

At foetal MRI the lesion (estimated diameters 45 × 11 mm) was situated above the diaphragm, close to the margin of the right cardiac chambers, presenting a low‐signal solid nodular inhomogeneous portion within a cystic component. No pericardial effusion was detected (Figure 2). The findings were compatible with a mass originating from the pericardium or the thymus.

The patient delivered vaginally at 37 + 3 weeks following pharmacological induction due to maternal–foetal isoimmunisation. A female neonate was born, in good clinical conditions, with a birthweight of 2980 g and an umbilical artery pH of 7.33. The newborn was admitted to NICU: cardiac sonography and CT scan were performed 10 days after birth, visualising the mass in the right para‐cardiac region, apparently originating from the thymus. Blood analysis showed serum alpha‐foetoprotein levels consistent with the patient's age (14 089 UI/mL); all other tested tumour markers (beta‐HCG, CEA, CA‐125, CA 19.9) were negative. These findings were suggestive of mature thymic teratoma.

**FIGURE 3:**
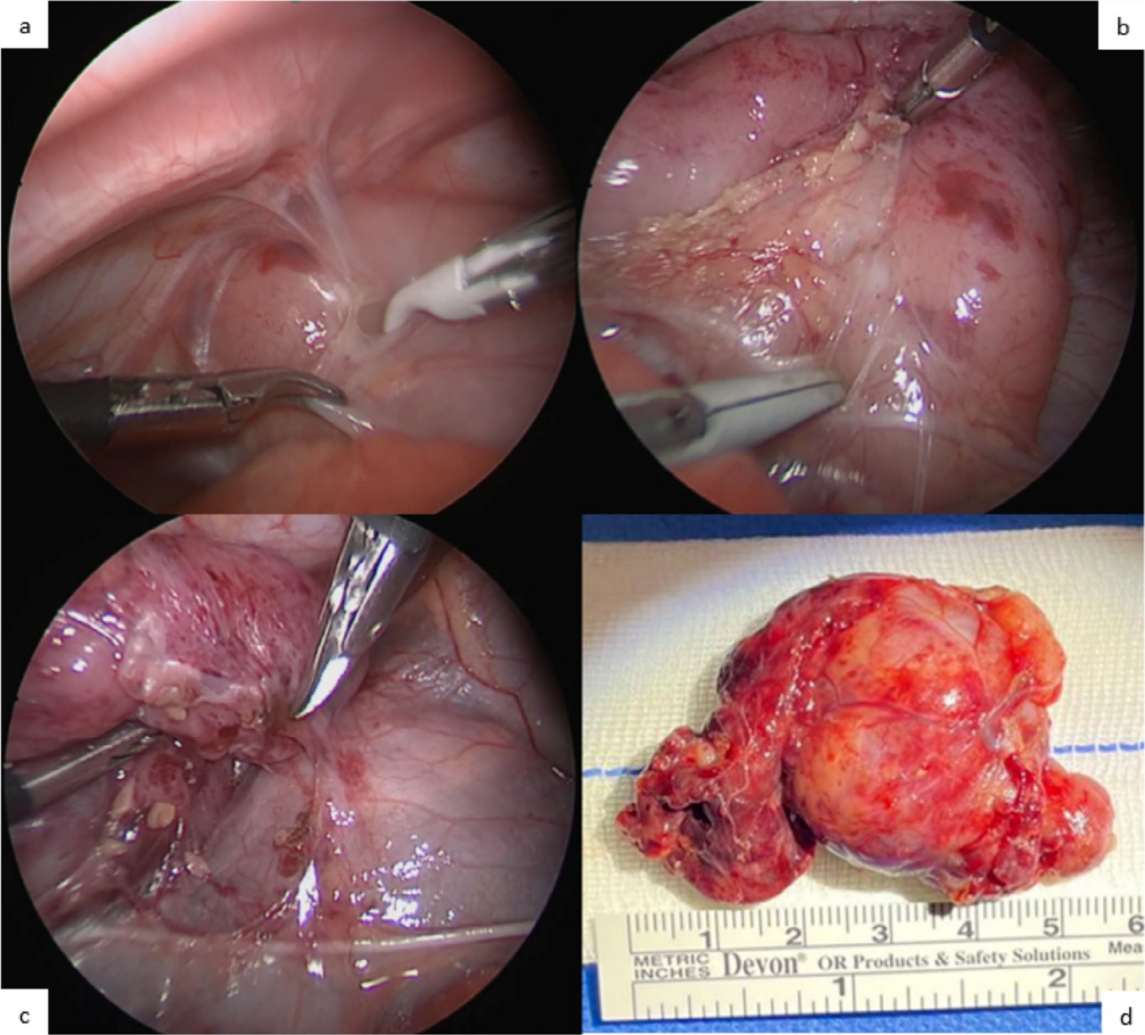
Thoracoscopic freeze frames showing (a) the mass at first inspection, (b) the opening of the pleura, (c) the gradual isolation of the mass. Finally, the excised mass and its dimensions are shown (d).

Due to the localisation of the mass, which posed a potential short‐term threat to the child's cardiorespiratory functions, VATS was performed 2 weeks after birth. General anaesthesia was induced and the patient was intubated with a 3‐mm‐ID orotracheal tube before being placed in left lateral decubitus. Surgery was then performed by two senior paediatric surgeons aided by a junior resident. A three‐port approach was employed, with a 5‐mm port placed on the right V intercostal space slightly medially to the anterior axillary line and two 3‐mm ports placed on the right VII intercostal space at the anterior axillary line and hemiclavear line, respectively. The mass was confirmed to originate from the thymus. It was carefully dissected starting from the apical portion by opening the mediastinal pleura and separating it from the mass, while carefully diving vessels supplying the upper thymic portion (Figure 3). A 3‐mm dissector, a 3‐mm JustRight bipolar sealer and 3‐mm scissors were used for these purposes. During the procedure, the left mediastinal pleura was accidentally opened, in the absence of intraoperative ventilatory or haemodynamic repercussions. For this reason, two thoracic tubes (one for each hemithorax) were left in place (ClassIntra grade II). Dissection was completed sparing a small part of the thymus. A mini‐thoracotomy was then performed by extending the 5‐mm trocar access incision on the V intercostal space up to a total length of 2 cm, after which extraction was achieved by gently tractioning the mass (whose consistency proved soft) through the widened incision.

Postoperative course was uneventful. The chest tubes were removed without complications, and the patient was progressively weaned from respiratory support (extubation on postoperative day 2). Parenteral nutrition, ongoing since the intervention, was gradually discontinued and full enteral feeding was achieved 8 days after surgery. The patient was discharged 2 weeks after the intervention.

At histological examination, a mature teratoma originating from the thymus (definitive measurements 7.0 × 3.5 × 2.0 cm) was confirmed, as well as its complete resection.

During follow‐up the patient remained in full health and experienced no recurrence of disease. Serum alpha‐foetoprotein level normalised 9 months after surgery. All other tumour markers remained negative for the duration of the surgical follow‐up (1 year after intervention).

## 3 Discussion

Mediastinal teratomas may pose a significant challenge because of their size and proximity to vital organs. Surgery is indicated to both establish the diagnosis and prevent life‐threatening complications caused by airway compression and haemodynamic compromise. Preoperative CT scan and MRI are paramount to evaluate the characteristics of the mass and its proximity to vital structures. An appropriate surgical approach must also consider other factors such as the patient's age, symptoms, comorbidities and cardiorespiratory status. Besides, the surgeon's experience and familiarity/preference for a specific technique play an important role.

In the available literature, open surgery is the preferred approach in most cases ranging from anterolateral/posterolateral thoracotomy, vertical incision along the midaxillary line, and clamshell incision to median sternotomy [3, 4]. Nevertheless, a safe dissection is not always easy to achieve due to the frequent presence of inflammatory adhesions, especially in cases of a ruptured teratoma [5].

VATS for removal of mediastinal masses has long been performed with excellent results in adults [6]. In children, it is considerably less performed than open surgery. Indeed, VATS for such rare lesions requires a level of surgical team expertise not easily attainable outside of high‐volume centres, and entails operating in sometimes very anatomically constrained spaces, especially in neonates. Additionally, although the validity of VATS is undisputed for most lung diseases in children, its indication for some paediatric tumours has been a matter of controversy in the past [7].

Sato et al. [8] described multiple cases of children with mediastinal tumours successfully treated with VATS. Mean age in children undergoing VATS was 6.9 ± 4.6 years and mean tumour size 53.1 ± 17.9 mm. Open resection was reserved in case of larger or malignant tumours, and in case of great vessels involvement or significant haemodynamic/respiratory compromise. Overall, VATS yielded no differences in postoperative and long‐term outcomes compared with open surgery. It was also associated with shorter thoracic drainage indwelling times and length of hospital stays. Although concerns have been raised in the past over the feasibility of VATS in mediastinal tumours larger than 5 cm, excellent results have been reported with bigger masses [9].

Robotic excision of mediastinal tumours has also been described. Zeng et al. [10] reported a series of 149 cases, 12 of which were thymic tumours, with a mean age of 5.9 years and masses greater than 5 cm in 84 cases.

To the best of our knowledge, ours is the only currently described case of successful thoracoscopic removal of a mediastinal mature teratoma in a newborn patient. VATS proved safe and effective in achieving complete resection without complications save from the accidental opening of the contralateral mediastinal pleura, which was managed conservatively by placing an additional chest tube. This result could be obtained even with a mass larger than 5 cm, which might be considered more significant taking into account the patient's age and dimensions. Although performing a 2‐cm mini‐thoracotomy was necessary to safely extract the whole mass, we believe its limited extension still proved advantageous over a traditional, wider thoracotomy or sternotomy in an open setting. By allowing for minimal rib spreading with sparing muscle technique, mini‐thoracotomies are known to enhance postoperative outcomes and lead to better cosmetic and functional musculoskeletal outcomes in the long‐term follow‐up. Thoracoscopy likely promoted a swift postoperative recovery, especially from a respiratory standpoint. In our case, this did not translate in a short length of stay: NICU protocols of our Institution dictated the necessity to perform full routine instrumental examination before ending hospitalisation, the execution of which required additional time even if discharge from a surgical point of view would have been feasible several days before.

In conclusion, VATS may be considered an effective approach for treating mediastinal teratomas, even in the presence of a large mass in a newborn, and will likely find increasing applicability in the future. However, such an approach should be carefully considered case by case, depending on a variety of tumoural factors and on the overall clinical status of the patient, as well as the experience and preferences of the surgical team.

## 4 Learning Points

- Mediastinal tumours may be safely and effectively excised by means of VATS even in very small children.
- VATS is not to be preferred over an open surgical approach a priori.
- Its applicability should be discussed on a case‐by‐case basis considering both tumoural characteristics and surgical team skills and preferences.

## Ethics Statement

The approval of the Institutional Review Board (IRB)/equivalent ethics committee of the Fondazione IRCCS Policlinico San Matteo, where the present case report was redacted, was not sought for this study, because the ethics committee of our institution deems that IRB approval is unnecessary for case reports that do not meet the DHHS definition of ‘research’ with anonymised data. The patient's parents provided written informed consent for the publication of the study data in anonymised form (being the patient a minor).

## Consent

The patient's parents gave their written informed consent for the use and publication of the patient's data in a manner that protects their identity and ensures confidentiality, provided that the abovesaid data would be used solely for scientific research purposes, and any identifying information would be securely stored and handled in accordance with privacy laws and ethical standards.

## Conflicts of Interest

The authors declare no conflicts of interest.

## Data Availability

The data that support the findings of this study are available on request from the corresponding author. The data are not publicly available due to privacy or ethical restrictions.
